# Systematic discovery of enzyme promiscuity in *Escherichia coli* using in vitro metabolomics

**DOI:** 10.1038/s42003-026-10099-x

**Published:** 2026-05-12

**Authors:** Christoph H. Gruber, Daniel C. Sévin, Elad Noor, Vito R. T. Zanotelli, Jennifer Schmitz, Nicola Zamboni, Uwe Sauer

**Affiliations:** 1 Institute of Molecular Systems Biology, ETH Zurich, Zurich, Switzerland; 2 PhD Program on Systems Biology, Life Science Zurich, Zurich, Switzerland; 3 Department of Plant and Environmental Sciences, Weizmann Institute of Science, Rehovot, Israel; 4 Division of Metabolism and Children’s Research Center, University Children’s Hospital Zurich, University of Zurich, Zurich, Switzerland

**Keywords:** Biochemical networks, Metabolomics, Enzymes

## Abstract

Metabolic enzymes have traditionally been regarded as highly specific catalysts; however, many can catalyze multiple reactions. To systematically investigate the prevalence of such enzyme promiscuity, we used nontargeted metabolomics to measure dynamic metabolite ion profiles in in vitro assays with 667 successfully purified *Escherichia coli* enzymes in a natural intracellular metabolome extract. Notably, nearly half of these enzymes elicited significant changes in ion traces. Using a machine learning-derived multivariate classifier at a false-discovery rate of 33%, we identified unexpected changes in 135 putatively annotated metabolite ion traces, indicating the presence of so far unknown promiscuous activities in 11% of the tested enzymes, most of which have yet to be recognized for their ability to catalyze multiple reactions. Notably, we found that nucleotide-related substrates or cofactors were enriched among the newly identified reactants. For 11 promiscuous enzymes, we successfully reconstructed 22 complete reaction stoichiometries, four of which were validated experimentally. Key findings include the nucleoside phosphorylase DeoA, for which we expanded the substrate range to include pyrimidines relevant to carbon and energy utilization, and the N-acetylmannosamine kinase (NanK), which displayed both cofactor and sugar substrate promiscuity. Additionally, CobC, a putative adenosylcobalamin/α-ribazole phosphatase, catalyzes flavin mononucleotide dephosphorylation, suggesting a generalist role in vitamin biosynthesis pathways. Beyond specific examples, the results suggest that metabolism harbors a wealth of underexplored catalytic flexibility, relevant for functional annotation, evolution, and genome-scale metabolic models.

## Abstract (web summary)

Metabolomics-based activity profiling of 667 purified *E. coli* enzymes reveals a surprising level of promiscuity, with many enzymes catalyzing unexpected reactions.

## Introduction

Metabolism has traditionally been viewed as a rigid network of specialized enzymes, each optimized for efficient and precise catalysis of a single biochemical reaction. However, increasing evidence challenges this perspective, suggesting that many, if not most, enzymes exhibit promiscuity ^1,2^. Enzyme promiscuity refers to the ability of enzymes to catalyze reactions beyond their primary evolved - or assumed - function. This includes the acceptance of a range of substrates or the catalysis of multiple distinct reactions^3–7^. These typically slower side activities are believed to be evolutionary remnants of ancestral enzymes that initially catalyzed diverse sets of reactions at low rates. Over time, certain functions were optimized through duplication and divergence, while traces of other functions linger as promiscuous activities, providing insight into the emergence of new enzymatic functions as well as whole pathways^7–9^. These latent metabolic functions have often been considered inconsequential under standard conditions, but in fact might confer fitness advantages when organisms encounter environmental stresses. Instead of evolving entirely new metabolic functions, even a single mutation can substantially enhance existing promiscuous activities, facilitating adaptation through the utilization of unexpected substrates or pathways^10–14^.

While primary reactions in metabolic networks have been thoroughly characterized through hypothesis-driven approaches, promiscuous reactions remain significantly underestimated. Current databases indicate that approximately 20% of enzymes in the well-studied *Escherichia coli* are known to catalyze more than one reaction^15^. Similarly, recent efforts to augment an *E. coli* genome-scale model with promiscuity data from existing databases have yielded a model featuring roughly 15% non-native reactions^16^. Identification of promiscuous enzyme activities is complicated by several factors, including their unexpected nature, order-of-magnitude differences in concentrations and reaction rates between native and promiscuous substrates, the masking of promiscuous activities by functionally redundant enzymes, and the tendency for these activities to manifest only under non-standard conditions^9,17–19^. Available methods for uncovering promiscuous activities include multicopy suppression screens^20,21^, picodroplet functional metagenomics^22,23^, and computational predictions based on genome-scale metabolic modeling^24^, sequence motifs^25,26^, or three-dimensional catalytic domains^27^. Although these studies typically identify a few dozen novel promiscuous reactions, their findings are modest when compared to substrate profiling studies, which are capable of testing extensive sets of substrate candidates^28–32^. Despite being limited to a few eligible enzymes, these profiling studies have frequently identified tens to hundreds of new substrates per enzyme, highlighting the substantial potential of comprehensive testing of a wide array of enzymes and potential substrates^33^.

Here, we aimed to systematically assess all known enzymes in *E. coli* with a wide range of potential substrates. To this end, we built upon a previously established method for identifying novel enzyme functions through endpoint metabolomics^34^. This method involves incubating purified proteins with metabolome extracts containing a diverse array of substrates, followed by the identification of depleted or accumulated metabolites as indicators of enzymatic activity. To better address the unique challenges of detecting promiscuous activities, we refined our approach by incorporating temporal resolution and integrated machine learning to develop multiparametric classifiers. With this enhanced methodology, we conducted broad-spectrum, untargeted activity profiling of 667 successfully purified metabolic enzymes in *E. coli*, using metabolome extracts as a diverse pool of substrates. With a 33% false-discovery rate, we identified 135 previously unknown promiscuous reactants corresponding to 76 distinct enzymes. From these, we reconstructed full stoichiometries for 22 previously uncharacterized reactions, four of which were validated experimentally.

## Results

### In vitro activity assays of all *E. coli* enzymes in the presence of metabolome extracts

For the systematic discovery of enzyme promiscuity in *E. coli*, we evaluated all known metabolic enzymes against a wide spectrum of potential substrates. We overexpressed all 1054 known metabolic enzymes in *E. coli*, using strains from the ASKA collection^35^. Each enzyme was purified via His-tag affinity chromatography in a 96-well format, resulting in biochemically detectable proteins for 667 enzymes (Supplementary Fig 1). To enhance the substrate coverage, we generated cofactor-supplemented, concentrated metabolome extracts from two distinct *E. coli* cultures: one grown in complex medium and the other in minimal medium, as previously described^34^. These extracts provided a rich source of diverse, naturally occurring candidate substrates.

**Fig. 1:**
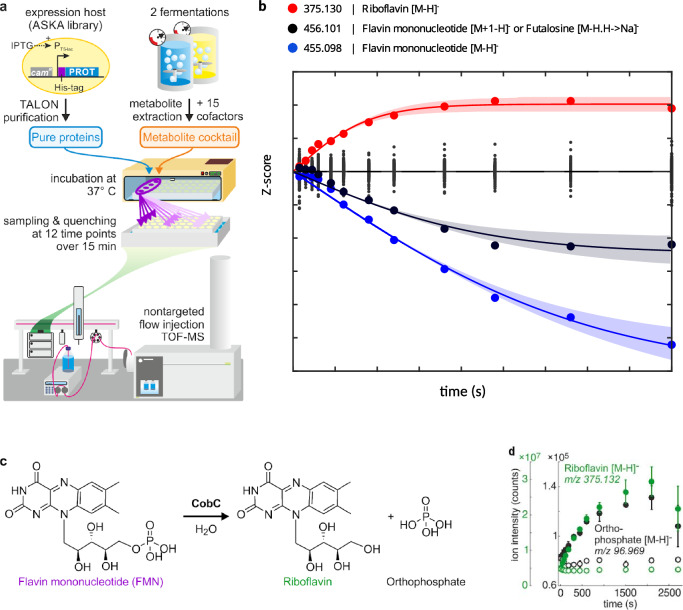
Discovery of enzyme promiscuity via non-targeted metabolomics. **a** Experimental setup. Proteins were expressed using strains from the ASKA collection^35^ and purified using TALON technology. The purified proteins were incubated in a concentrated metabolome extract prepared from *E. coli* cells grown in two different fed-batch fermentations, supplemented with fifteen general enzyme cofactors, including ATP and NADPH. Over a duration of 15 min, twelve aliquots of the reaction solutions were collected and quenched in methanol at −20 °C. Metabolite levels were then measured using nontargeted flow injection mass spectrometry^37^. **b** Time courses of metabolite ions. The time courses of 9554 metabolite ions in the enzyme assay of CobC. Data points from three ions are highlighted, with a solid line representing a least-squares fit using a hyperbolic tangent (sigmoid) function. Shaded areas denote the 95% confidence intervals calculated from parameter estimates. **c** Reaction formula. The reaction formula of the CobC-catalyzed dephosphorylation of flavin mononucleotide (FMN). **d** Experimental validation. Validation of the dephosphorylation of FMN through incubation with purified CobC. Additional experimental data for CobC validation are in Supplementary Fig 4.

Subsequently, each enzyme was incubated in these extracts, and we monitored the time courses of 9,554 detectable mass-to-charge (*m/z*) features - essentially metabolite ions - during each enzyme assay using nontargeted flow injection time-of-flight mass spectrometry (Fig. 1a). Among these, 411 ions were putatively annotated as 791 unique metabolites, achieved by matching the measured accurate *m/z* values with the monoisotopic masses of compounds in the KEGG *E. coli* metabolite database (Supplementary Data 1)^36^. This generated a dataset equivalent to over half a million single-substrate enzyme assays, though it comes with the inherent limitations of being unable to differentiate between isomers with identical formulas or to account for potential in-source fragmentation^37^. Following the correction for technical biases such as instrument drift and batch effects (Supplementary Fig 2), we determined the relative abundances of the 9,554 detectable metabolite ions. To correct for systematic degradation and impurities, we normalized the data, adapting a previous approach^34^ by iteratively calculating bi-directional Z-scores (hereafter simply referred to as Z-score in this manuscript) for each ion in each enzyme assay. This process was performed first relative to each ion’s abundance in all other enzyme assays and time points, and then relative to the abundance of all other ions in the same enzyme assay, repeated five times.

Ion profiles obtained for each enzyme are exemplified by Fig. 1b, which shows the time-course profiles of all detected metabolite ions in the CobC assay. CobC has been annotated as a multispecific α-ribazole phosphatase and adenosylcobalamin phosphatase based on its high sequence homology with *Salmonella typhimurium* CobC^38^. Notably, we did not observe any catalysis of its currently annotated reactions; this is, in part, due to the putative reactants for the adenosylcobalamin phosphatase reaction being outside the detectable *m/z* range. Instead, our findings revealed a depletion of metabolite ions annotated as flavin mononucleotide (FMN) and an accumulation of metabolite ions corresponding to riboflavin. From this observation, we hypothesized that CobC may be promiscuously catalyzing the FMN phosphatase reaction (EC 3.1.3.102) (Supplementary Fig 3). We confirmed this by incubating purified CobC with pure FMN, upon which we could detect an increase in metabolite ions corresponding to riboflavin and phosphate (Fig. 1c, d).

### Random forest integrates dynamic ion trace features for discovery of novel reactants

To systematically identify putative substrates and product ions, we derived metrics that capture the dynamic behavior of metabolite ions in each enzyme assay and used them as inputs in a random forest machine learning model. Among the features we analyzed were the average Z-score throughout the entire time course, along with the average Z-score and geometric mean rank product over the last five time points, which was when most reactions were anticipated to reach equilibrium. The Z-scores quantified the response magnitude in standard deviations, while the rank products indicated consistency across assays^39^. Leveraging the dynamic nature of our data, we also fitted a hyperbolic tangent (sigmoid) function to the metabolite ion time courses. This allowed us to estimate the Z-score at equilibrium (parameter *A* in Eq. 4) and its steepness (parameter *B* in Eq. 4). The goodness-of-fit for these curve fits was evaluated using the mean squared error (MSE). This comprehensive, multivariate feature set was subsequently used to train a random forest classifier aimed at identifying substrates and products of enzymatic activity.

**Fig. 2:**
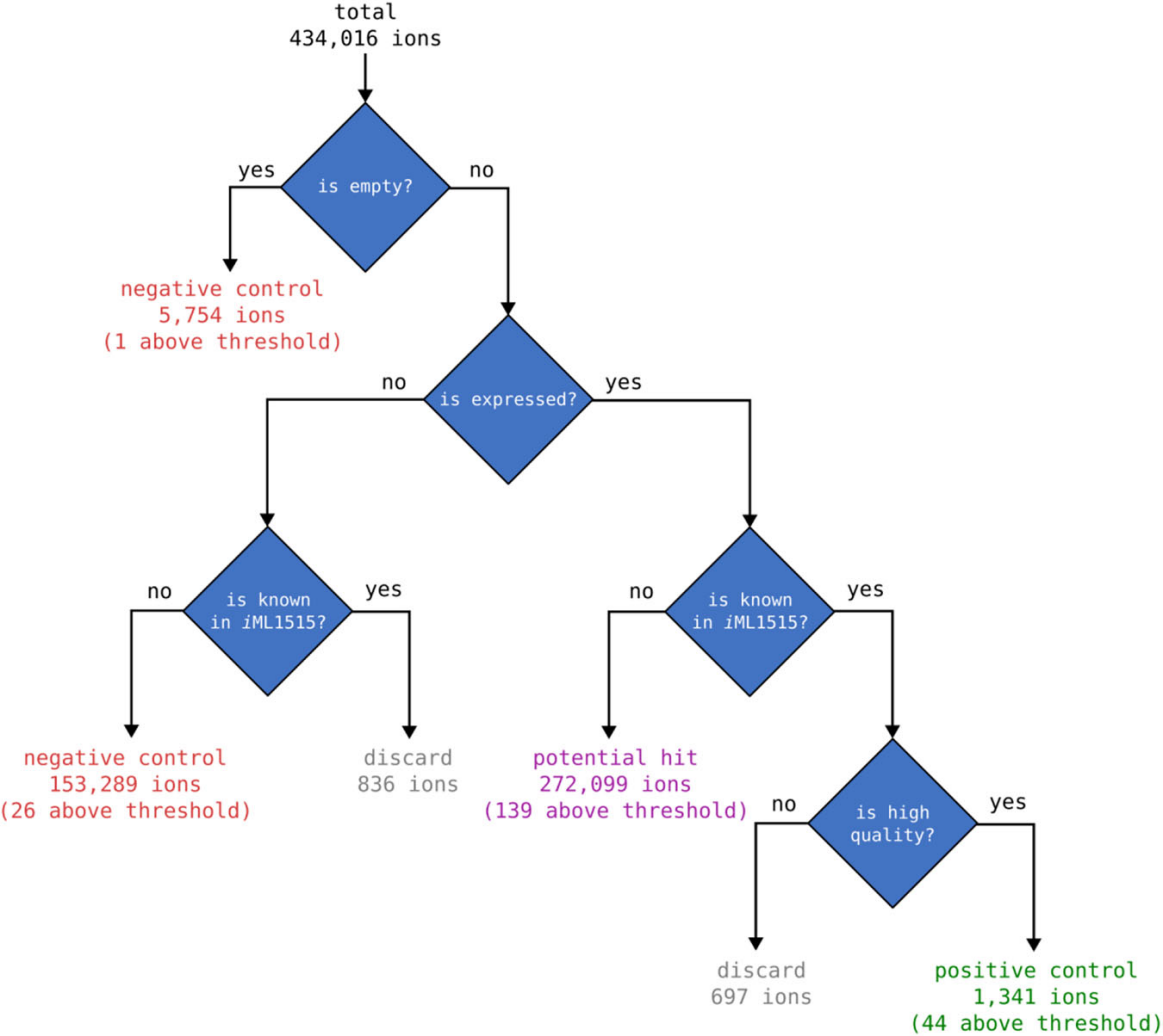
Decision tree for assigning ions to training classes. Depicted is a flowchart illustrating how 434,016 total measured ion time courses were assigned to different categories based on whether or not purified enzyme was added to the assay mixture (is empty?), whether the enzyme’s expression was detectable (is expressed?), whether the ion is a known reactant (is known in iML1515?), and whether at least one annotation matched the deprotonated form [M-H]^-^ of a carbohydrate (is high quality?). The annotation “above threshold” indicates the number of ions that were above the 33% false discovery rate threshold in the random forest model described in Fig. 3.

For training, we assigned ion time courses into classes to serve as input for machine learning (Fig. 2). We defined negative control ions as ions of assays in which either no enzyme was added, or the expression of the added enzyme was below the detection limit. Since there was still a chance for some low activity in the latter case, we conservatively excluded the known reactants of these added enzymes from the negative control, leaving n_neg = 159,043 ions for the training phase. To compile a list of positive control ions, we compared the detected ions of each assay with the set of known reactants based on the iML1515 metabolic model of *E. coli*^40^. An ion was classified as a positive control ion – indicating a previously known reaction – if at least one of its possible annotations matched a known reactant (n_pos = 1341). It is important to note that only ions annotated based on the deprotonated form ([M-H]^-^) of metabolites were considered during training. Ion traces corresponding to metabolite adducts (e.g., ¹²C → ¹³C or H^+^→Na^+^) were excluded, as they would typically be much less abundant and less informative than the deprotonated form of the same metabolite.

**Fig. 3:**
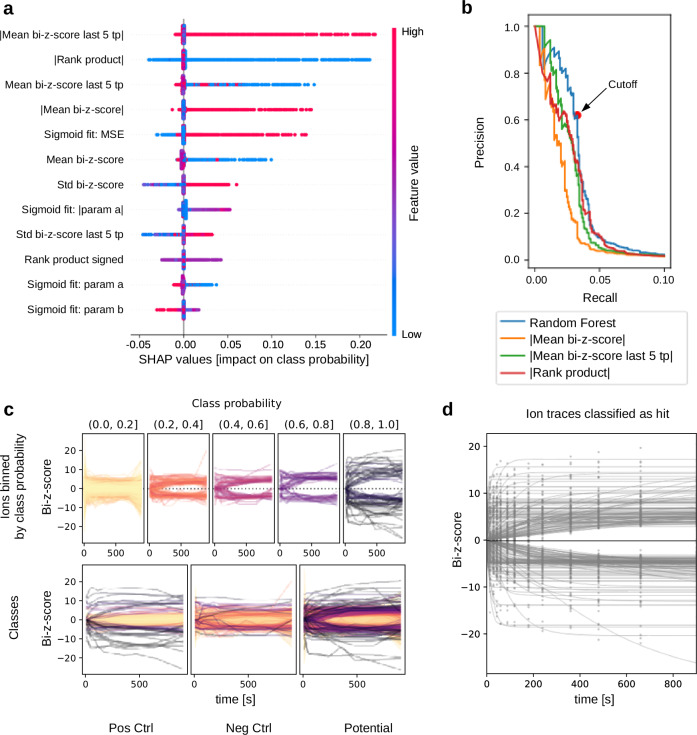
Identifying enzymatic reaction hits based on dynamic ion trace features with random forest classifier. Individual features were extracted from ion traces, and the classifier was trained using ion traces annotated according to the deprotonated mass of metabolites. Training was done based on known reactants (Pos Ctrl) and negative control ions (Neg Ctrl) **a** SHAP values representing an estimated impact of each feature for the class prediction of the trained random forest classifier. **b** Precision-recall analysis. Precision-recall curves of random forest predictors are compared with the most important individual features. The red dot highlights the cutoff in the random forest probabilities corresponding to a 33% false-discovery rate. **c** Raw ion traces by class probability. At the top, examples of raw ion traces are shown and grouped by class probability, with each group assigned a different color. At the bottom, the classes used for training are displayed. Ion traces corresponding to known reactants are categorized as positive control ions (Pos Ctrl), while ion traces from assays without added enzymes serve as negative control ions (Neg Ctrl). The remaining ion traces indicate potential novel activities (Potential). All raw ion traces are colored according to their class probability. **d** Classification of hits. Plot of all ion traces classified as hits based on the random forest classifier. Points correspond to raw Z-scores, while curves represent a least-squares fit based on hyperbolic tangent (sigmoid) function.

Next, we trained a random-forest classifier on ~160,000 labeled ion time traces (Methods). The final model comprised 10,000 trees, each limited to a depth of five, and randomly sampled 20% of the 12 features per tree. The performance metrics of the out-of-bag predictions on the training set were comparable to the test set, indicating little overfitting (accuracy out-of-bag and test-set error both 0.997, F1-score: training out-of-bag: 0.044, test set: 0.056). Finally, the classifier was retrained with the selected parameters and the full dataset. For downstream analysis, the probability of a sample being classified as a positive control was used as a predictor. We used SHapley Additive exPlanations (SHAP) values to analyze the influence of each feature on the predicted probabilities. The features with high SHAP values were generally derived from the average Z-scores and rank product, thus suggesting that response consistency and magnitude were the most informative for classification (Fig. 3a). From the curve-fitting features, the goodness-of-fit parameter MSE but not the actual fitted parameter values (sigmoid fit: param a/b) had a substantial influence on some of the prediction results (SHAP value > 0.05, Fig. 3a). To see how well each feature would be able to serve as a classifier on its own, we performed precision and recall analyses for individual features and found that they were not sufficiently discriminative and clearly outperformed by the multivariate random forest classifier (Fig. 3b).

The trained classifier demonstrated its ability to effectively distinguish substrate- and product-like ion traces from background noise. This was particularly evident when we grouped raw ion traces by their predicted probabilities to be similar to positive controls (class probabilities); time courses exhibiting high Z-scores and well-defined sigmoid-like saturation curves were consistently assigned high probabilities, whereas lower amplitude traces received very low probabilities (Fig. 3c). For the identification of putative novel substrates and products, we established a threshold corresponding to an estimated false discovery rate of 33%, a cutoff chosen for discovery and corresponding to a positive-class probability of 0.35. This threshold means that we expected 33% of the declared hits to be false positives in the final predictions. This balances precision (0.61) and recall (0.03) among the positive and negative controls, maintaining an estimated false positive rate of 0.00017 (Fig. 3b). The low recall indicates that even when using machine learning most known reactants (positive controls) were indistinguishable from noise. With this threshold, among the deprotonated ions alone, 183 ion traces were classified as hits (Supplementary Data 2). In contrast, applying the same cutoff to ions corresponding to metabolite adducts or unannotated ion traces would result in over a thousand total hits, albeit at a much higher false-discovery rate (FDR = 0.90, Supplementary Data 3). None of the known reactants in the assays with non-detectably expressed enzymes were found to be above the threshold, supporting a lack of enzymatic activity in these assays. A comparison of all ion traces declared as hits using this threshold underscores the classifier’s effectiveness, as lower amplitude traces with weak or inconsistent signals were successfully filtered out (Fig. 3d).

### At least 11% of screened enzymes affected unexpected metabolite ions

**Fig. 4:**
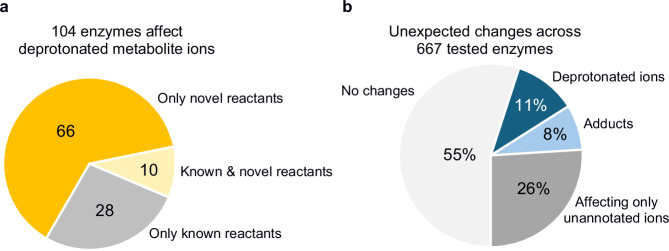
Catalysis of reactions with previously unknown reactants in at least 11% of *E. coli* enzymes. **a** Enzymes affecting known vs. novel reactants. The numbers of enzymes affecting known and novel reactants, classified by machine learning classifier based solely on high-confidence ion traces at a false-discovery rate of 33%. **b** Unexpected changes in ion traces. The percentage of enzymes exhibiting unexpected changes in ion traces when considering only high-confidence ions (i.e., deprotonated ions ([M-H]^-^), along with those considering adducts (e.g., ¹²C → ¹³C or H^+^→Na^+^) and including unannotated ions.

To determine how many of the 667 detectable enzymes catalyzed promiscuous reactions, we initially concentrated on ion traces classified as high-confidence hits, i.e., those annotated based on the deprotonated form. We compared these annotations to known enzymatic reactions cataloged in the iML1515^40,41^ model of *E. coli*. If a metabolite ion received an annotation that did not match any known reactant, we classified it as a novel reactant (Supplementary Data 2 and 3). As an additional validation step, we cross-referenced our list with the KEGG database^36^, identifying four additional matches to previously reported reactions. These were excluded from the set of novel reactants. We found that approximately 6% of the purified enzymes exhibited activity consistent with known reactants, translating to a total of 48 known reactants detected across 38 enzymes. In contrast, the remaining 94% of enzymes did not show any known catalytic activity under our conditions. This lack of observed activity could be attributed to several factors, such as the purified enzymes being inactive or inhibited by compounds present in the metabolome extract, or the reaction rate going below the detection limit due to insufficient substrate concentrations and/or thermodynamic driving forces. Importantly, we identified 135 metabolites ion traces across 76 enzymes that were not previously annotated as reactants. Among all 104 enzymes that we identified to affect metabolite ions, 28 enzymes affected exclusively known reactants, 10 enzymes displayed a mix of known and so far unknown reactants, and 66 enzymes showed exclusively novel reactants (Fig. 4a). Overall, 76 enzymes—representing 11% of tested enzymes—were assigned novel reactants.

Among the 76 enzymes with unexpected reactants, approximately 40% (29 enzymes) were previously recognized for catalyzing multiple reactions, while over half (46 enzymes) had been considered specific and would now be reclassified as promiscuous according to our findings. Notably, in the case of the enzyme YsgA, no reaction had been assigned previously (Supplementary Data 4). Consequently, our results based on high confidence annotations support the notion that enzyme promiscuity is a prevalent characteristic.

We then investigated how including lower-confidence annotations, such as metabolite adducts and unannotated ions, would influence these findings. While deprotonated ions typically yield higher-quality data, there are instances where adducts could provide valuable insights, particularly in cases where the deprotonated form overlaps with another ion peak. Unannotated ions, frequently encountered in untargeted metabolomics, may result from in-source fragmentation or chemical reactions but could also signify yet-uncharacterized metabolites that might hold significance in future studies^42^. When evaluating both higher- and lower-confidence annotations for unexpected changes, we identified a total of 1084 enzyme-ion pairs as hits, corresponding to an average of less than four differential ions per affected enzyme. This resulted in 702 unique ion hits across 299 enzymes, averaging 2.3 unique ions per enzyme. Therefore, even with the inclusion of lower-confidence annotations, the enzyme-reactant pairs appeared to remain specific. Overall, including adduct ions roughly doubles the proportion of enzymes associated with unexpected metabolites from 11 to 19%, while incorporating unannotated ions further increases this to 45% (Fig. 4b). It is important to note that while the 33% false discovery rate applies solely to deprotonated ions and would be much higher when considering all ions, these results imply that the 11% of previously unknown promiscuous enzymes identified from deprotonated ions alone is a conservative estimate. Although our preliminary exploration of adducts and unannotated ions suggests that metabolic promiscuity may be more widespread and underscores the potential of our dataset as a resource for future studies, we focus the remainder of our analyses on only high-confidence annotations to ensure robustness and enhance interpretability.

### Enrichment analyses reveal overrepresentation of nucleotides

To investigate patterns among the discovered promiscuous reactions, we conducted pathway enrichment analyses focusing on high confidence annotated reactants. Instead of comparing against the full *E. coli* metabolic network, we used all annotated ions detected in our assay as the background, thereby focusing on pathway preferences within the measurable metabolite space. In this approach, purine metabolism emerged as the only pathway significantly enriched after multiple-testing correction, while other nucleotide-related pathways (nucleotide sugar metabolism, riboflavin metabolism, pyrimidine metabolism) showed nominal signals but were not significantly enriched (Supplementary Data 5). This suggests an overrepresentation of nucleotides among the unexpected reactants, which may be attributed more to methodological factors rather than inherent features of enzyme promiscuity. The polar extraction procedure used to prepare the metabolite cocktail favored pathways with polar intermediates. Additionally, cofactors such as triphosphate nucleotides were added at high concentrations to ensure they were not limiting.

Furthermore, we executed an enzyme-centric pathway enrichment analysis for all enzymes exhibiting unexpected reactant changes. In contrast to the metabolite-centric analysis, the distribution of enzymes was broadly spread across pathways with no significant enrichment. This suggests that promiscuous activities are not strongly clustered in certain pathways and that the observed distribution is unlikely to be driven by methodological bias. Nevertheless, larger datasets or modifications to the presented setup may reveal patterns that remained undetectable here.

### Inference of full reaction stoichiometries for subset of enzymes

To infer biochemical reactions from the observed metabolite changes, we employed computational approaches inspired by previous work (Supplementary Fig 3)^34^. Our initial aim was to conservatively identify reactions and facilitate the discovery of enzymes that catalyze mechanistically distinct reactions by matching detected substrate and product metabolites with the KEGG main reactant pair database. Additionally, we sought to account for enzyme promiscuity, wherein enzymes can catalyze multiple reactions that share one or more substrates or products (including cofactors). To identify such cases, we selected KEGG main reactant pairs that included a detected novel substrate or product metabolite in conjunction with a known reactant. From these observations, we reconstructed complete reaction stoichiometries for 22 unique reactions across 11 enzymes (overview in Table 1, full information in Supplementary Data 6 and 7). Notably, five enzymes – including NanK and FolE – were previously reported to catalyze only a single reaction, while the remaining six enzymes, such as DeoA, were already recognized as exhibiting substrate promiscuity, further expanding their repertoire of catalyzed reactions with our findings. Aligning with the results from metabolite-based pathway enrichment, the reconstructed stoichiometries often involved cofactors, particularly nucleotides like ADP and ATP. This was especially true for those reactions predicted based on declaring both product and substrate as hits in our screen, implying that a substantial proportion of these reactions revolve around nucleotide interconversions or the use of nucleotides as phosphate donors. Also noteworthy is some redundancy in the predicted reaction substrates and products, likely caused by MS1 ambiguity leading to an overestimation of reactions. For example, from both Glk and NanK assays, separate reactions were reconstructed using either ADP or adenosine-3’,5’-bisphosphate as a substrate. However, since these metabolites have identical mass (*m/z*) and are therefore indistinguishable in our MS1-based annotation, it is likely that only one of these reactions is occurring in the assays.

**Table 1:** Full reaction stoichiometries for a subset of 11 enzymes

Enzyme	Newly predicted reaction			[EC]	Promiscuity
CobC	FMN + H_2_O	→	riboflavin + phosphate	[3.1.3.102]	Substrate promiscuity
Glk	ADP + H_2_O	→	IDP + ammonia	[3.5.4.7]	Catalytic promiscuity
	ADP + dGDP	→	ATP + dGMP	[2.7.4.8]	
	Adenosine 3’,5’-bisphosphate + H_2_O	→	AMP + phosphate	[3.1.3.7]	
	dGTP + H_2_O	→	dGMP + diphosphate	[3.6.1.9]	
	ATP + GTP	→	AMP + pppGpp	[2.7.6.5]	
	UTP + AMP	→	UDP + ADP	[2.7.4.10]	
	ADP + GTP	→	ATP + GDP	[2.7.4.6]	
NanK	dGTP + H_2_O	→	dGMP + diphosphate	[3.6.1.9]	Catalytic promiscuity
	ATP + GTP	→	AMP + pppGpp	[2.7.6.5]	
	2 ADP	→	ATP + AMP	[2.7.4.3]	
	ADP + dGDP	→	ATP + dGMP	[2.7.4.8]	
	Adenosine 3’,5’-bisphosphate + H_2_O	→	AMP + phosphate	[3.1.3.7]	
	ADP + H_2_O	→	IDP + ammonia	[3.5.4.7]	
RstB	Deoxyadenosine + H_2_O	→	deoxyinosine + ammonia	[3.5.4.4]	Catalytic promiscuity
AcpS	2 Acetyl-CoA	→	CoA + acetoacetyl-CoA	[2.3.1.9]	Substrate promiscuity
DeoA	Uridine + phosphate	→	uracil + ribose 1-phosphate	[2.4.2.3]	Substrate promiscuity
Epd	erythrose 4-phosphate + xylulose 5-phosphate	→	fructose 6-phosphate + glyceraldehyde 3-phosphate	[2.2.1.1]	Catalytic promiscuity
FolD	2-amino-5-formylamino-6-(5-phospho- ribosylamino)pyrimidin-4(3H)-one + H_2_O	→	2,5-Diamino-6-(5-phospho-ribosylamino)pyrimidin-4(3H)-one + formate	[3.5.1.102]	Catalytic promiscuity
FolE	Formyl-glutamate + H_2_O	→	formate + glutamate	[3.5.1.68]	Substrate promiscuity
MalP	UDP-glucose + galactose 1-phosphate	→	glucose 1-phosphate + UDP-galactose	[2.7.7.12]	Substrate promiscuity
TyrA	erythrose 4-phosphate + xylulose 5-phosphate	→	fructose 6-phosphate + glyceraldehyde 3-phosphate	[2.2.1.1]	Catalytic promiscuity

Listed are the obtained reaction stoichiometries for a subset of enzymes, derived from matching the 204 identified reactant hits with entries in the KEGG reaction pair database. The following rules were applied: (i) identification of substrate and product that match a reaction pair (CobC–RstB), (ii) Detection of reactants and previously known reactants that correspond to the substrates and products of the identified reaction pairs (AcpS–TyrA). The stoichiometries listed were formulated based on the resulting set of reaction pairs. The shown reaction arrow is one-sided, but reversibility remains undetermined and requires experimental validation. Also shown is the promiscuity type (substrate or catalytic) for both known and identified reactions. If shown in bold, the identified reaction(s) changed the previous type, whereas gray indicates it is identical to the previous one. A more comprehensive table with additional details, including known reactions, is available in Supplementary Data 6 and 7.

To differentiate between enzymes that exhibit substrate ambiguity and those with genuine catalytic promiscuity, we conducted a comparative analysis of the mechanisms associated with known and predicted promiscuous reactions (Supplementary Data 7). For two of the five enzymes previously classified as specific, for which we could reconstruct full stoichiometries of promiscuous reactions, the newly predicted reaction suggests substrate promiscuity, meaning the novel reaction is similar to the known one but involves different substrates. The remaining three predictions suggest catalytic promiscuity, as indicated by a different first digit in the EC number, pointing to a shift in catalytic mechanism rather than just substrate flexibility. Thus, our dataset reveals both substrate and catalytic promiscuity, demonstrating its ability to detect functionally distinct reactions that may connect distant pathways.

### Experimental validation of selected promiscuous enzymes

To validate the predicted promiscuous reactions, we selected a subset for functional experiments based on the availability of substrates and their functional relevance. The purified enzymes were incubated in an enzyme buffer containing only the predicted substrates and cofactors. In certain cases, substrates structurally similar to known or predicted reactants were also examined to assess potential substrate promiscuity. The readout of these enzyme assays was done by mass spectrometry to determine whether the expected products were formed.

**Fig. 5:**
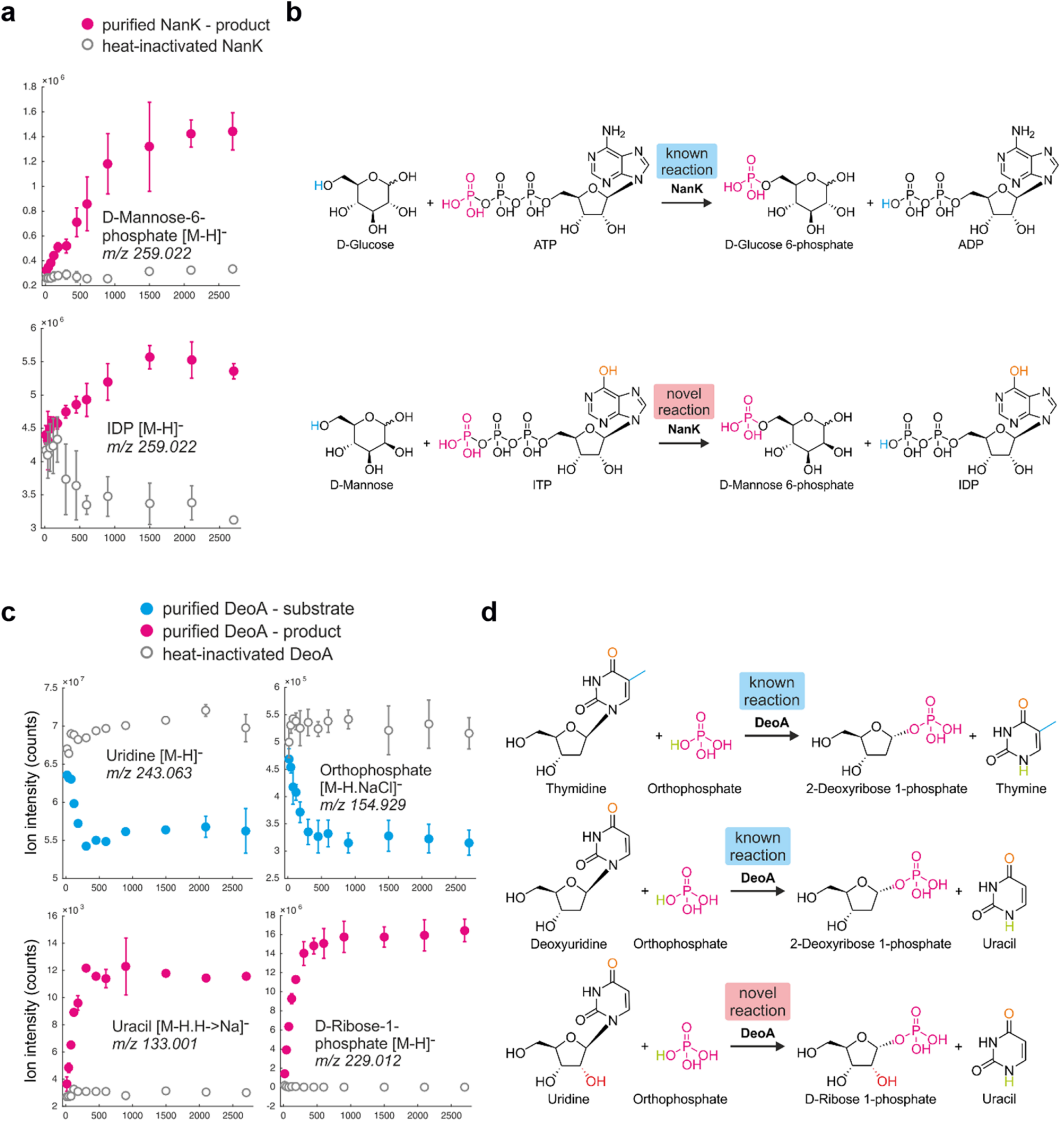
Experimental validation of previously unknown promiscuous activities. **a** Validation of NanK. Ion traces obtained from mass spectrometry-based validation experiments, showcasing the incubation of purified NanK in assay buffer containing only the substrates D-Mannose and ITP. **b** Reaction schemes of NanK. Depiction of known and novel reaction schemes of NanK. **c** Validation of DeoA. Ion traces of supplied substrates and expected products upon incubation with purified DeoA. **d** Reaction schemes of DeoA. Depiction of known and novel reaction schemes of DeoA.

The first enzyme tested was CobC, which is annotated by similarity as a phosphatase acting on cobalamin intermediates in vitamin B12 biosynthesis. These canonical substrates are larger than the m/z 50–1000 window used in our FIA-MS analysis and could therefore not be detected. Instead, we confirmed the predicted dephosphorylation of FMN to riboflavin (Fig. 1b–d, Supplementary Fig 4). Although the mechanisms of the annotated and validated reactions are similar, our results demonstrate CobC activity on a chemically distinct substrate, suggesting a generalist role in vitamin biosynthesis. Next, we investigated N-acetylmannosamine kinase (NanK), which has been characterized to phosphorylate N-acetyl-D-mannosamine to N-acetyl-D-mannosamine 6-phosphate with ATP as a phosphate donor. As predicted, NanK exhibited additional inosine triphosphate (ITP)-dependent kinase activity, converting D-mannose into D-mannose 6-phosphate and producing inosine diphosphate (IDP) (Fig. 5a), thereby showcasing cofactor promiscuity (Fig. 5b). Furthermore, our tests of NanK incubated with D-fructose – due to its structural similarity to the main substrate D-mannose - revealed additional sugar substrate promiscuity (Supplementary Fig 5). Next, we examined the purine deoxynucleoside phosphorylase DeoA, which is already known to catalyze multiple reactions, including the utilization of pyrimidine deoxyribonucleosides as carbon and energy sources. We confirmed that DeoA can utilize alternative substrates such as uridine (Fig. 5c) and the structurally similar cytidine (Supplementary Fig 6). This finding expands the known substrate range of DeoA (Fig. 5d), broadening the set of potential energy and carbon sources it can utilize.

Lastly, we examined the pyrimidine-specific ribonucleoside hydrolase RihB, known to catalyze the hydrolysis of uridine and cytidine. Unlike the previous validations, we based these experiments on the results from a low-confidence ion trace; specifically, a potassium thymidine adduct suggesting thymidine might be a potential substrate. When testing this potential new substrate and other compounds that are structurally similar to the known substrates, we found that RihB is additionally capable of acting on thymidine, guanosine, and deoxyuridine as substrates (Supplementary Fig 7). This finding not only expands the known substrate range of RihB but also highlights the potential for uncovering additional enzymatic activities among the low confidence hits we reported.

## Discussion

Metabolic enzymes have long been regarded as highly specific catalysts, yet systematic experimental confirmation for this assumption has been lacking. Through the successful purification and testing of 667 metabolic enzymes from *E. coli* for promiscuous catalytic activity towards hundreds of candidate substrates using untargeted metabolomics, we identified 76 enzymes that acted on metabolites not involved in their previously annotated reactions. Upon expanding our analysis to include ion traces with less confident annotations - such as metabolite adducts and unannotated ions, albeit at a much higher expected false-discovery rate - we discovered that close to half (299) of the tested enzymes led to significant changes in ion traces. Furthermore, we determined complete stoichiometries for 22 of these previously unknown promiscuous reactions, successfully verifying four experimentally.

A limitation of our approach is that in vitro enzyme activities might not reflect those in living cells due to potential absence of post-translational modifications that ensure specificity or the need for interaction with other proteins. Furthermore, as we were unable to quantify absolute reaction rates, no claims can be made regarding the observed rates in identified reactions. Nevertheless, in contrast to most other methods for discovering enzyme promiscuity, the presented approach provides direct, experimental, and data-driven evidence for catalytic activity. By employing untargeted metabolomics-based enzyme assays within complex metabolome extract, we mitigate biases that stem from assumptions about potential promiscuous substrates and detect activities even if they do not involve a metabolite that is detectable by absorbance-based assays. However, this approach has inherent limitations: it is blind to reactions that interconvert metabolites with the same mass (such as isomerases), ions outside the range of 50–1000 Da, and reactions that are already at or near equilibrium, which may appear indistinguishable from noise. These limitations imply that the actual number of promiscuous reactions is likely much higher.

Identifying reactants from the thousands of noisy ion measurements poses an analytical challenge: Known reactants account for only 0.045% of all measured ions, requiring a high specificity to confidently identify reactants - estimated in the form of false-discovery-rate. This study addressed this challenge in several ways: Using time-course measurements instead of end point measurements, it was attempted to use the reaction kinetics’ shape to improve discriminatory power. Indeed, using machine learning to leverage such multivariate readouts enabled us to identify ions with higher confidence than univariate thresholding (Fig. 3b). A major challenge in training the classifier was the lack of a separate training data set. We wanted to report recovery of known reactants while using them as well as a positive set for training. This was achieved by leveraging the out-of-bag predictions, a unique property of random forests, to provide predictions based on trees of the classifier not trained on the training data^43^. These predictions are known to have a slightly higher error, as they are based on about one third of the trees and thus represent a conservative estimate^44^. Finally, the low recovery rate of known reactant ions (ca 3%) - consistent with a manual, visual inspection of the data - posed an additional challenge to train the classifier. This exacerbated the existing class imbalance between positive and negative controls, as most known reactants were effectively indistinguishable from random negative control ions.

Among our key findings are the cofactor and substrate promiscuity of NanK and the expanded substrate range of DeoA, a known purine phosphorylase that we identified to also act on uridine and cytidine, expanding its substrate range within nucleotide salvage pathways. Additionally, CobC, annotated by similarity as a phosphatase acting on cobalamin intermediates in vitamin B12 biosynthesis, was found to dephosphorylate FMN, suggesting a broader role in nucleotide or cofactor metabolism. These examples provide insights into the flexibility of metabolism, with promiscuous enzymes being able to catalyze reactions in distinct pathways and other promiscuous activities allowing cells to tap into diverse sources of alternative substrates when primary substrates are scarce. Beyond specific examples, the broader trends suggest that metabolism harbors a wealth of underexplored catalytic flexibility, relevant for both genome-scale metabolic models and functional annotations. We emphasize that the many detected reactions involving non-annotated metabolite ions are not considered in our analysis, and that our growing knowledge of the *E. coli* metabolome may affect our conclusion.

The large number of identified activities involving non-annotated ions – currently lacking biochemical interpretation – may prove increasingly relevant. As the array of annotated metabolites continues to grow, our data set can be revisited to uncover further orphan enzyme functions from presently non-annotated ions. This expanding set of unannotated ions could signify previously overlooked metabolites that play crucial roles in processes like stress responses, signaling, or other regulatory functions. Some of these unannotated ions might also stem from undesired side activities that convert useful intermediates into ineffective or even toxic waste products. Consequently, promiscuous reactions could aid in alleviating what is termed “metabolite damage”. Evidence for such mechanisms has been documented in other studies, suggesting that promiscuous enzymes may fulfill dual roles in promoting metabolic flexibility and damage mitigation^45^. Additionally, unknown metabolites may belong to unexplored metabolic pathways that have evaded traditional laboratory analyses. This notion is supported by the substantial number of phylogenetically conserved, functionally uncharacterized genes which, even in the case of *E. coli*, comprise over 30% of the genome^46,47^.

In summary, our study illustrates that enzymatic promiscuity is a widespread characteristic across *E. coli*’s metabolism. The fact that many known enzymatic reactions remained undetected in our assays suggests that a substantial fraction of promiscuous activities also went unnoticed. Thus, the true extent of metabolic promiscuity is likely even larger than our findings reveal.

This work represents a step towards the comprehensive mapping of metabolic stoichiometry. We believe that our dataset will serve as a foundation for various future studies, including elucidating the biological roles of specific promiscuous enzymes, assigning functions to currently unknown genes, and further investigating the significance of promiscuity on a genome scale.

## Methods

### Media and bacterial cell cultivation

All *E. coli* strains used in this study were obtained from the ASKA collection^35^. These strains were derived from *E. coli* K-12 AG1 and harbored a plasmid, pCA24N, which confers chloramphenicol resistance and carries a His_6_-tagged open reading frame under the control of an isopropyl-β-D-thiogalactoside (IPTG)-inducible T5-lac promoter. Cultures were inoculated from glycerol stocks stored at −80 °C and were cultivated in LB medium (10 g/L Bacto tryptone, 5 g/L Bacto yeast extract, 5 g/L NaCl, pH 7.4), supplemented with 100 µM IPTG and 100 µM chloramphenicol, for 16 h at 37 °C while shaking at 300 rpm. Screening was conducted in 96-deep well plates, with 1.5 mL medium per well. For validation experiments, we used 500 mL baffled shake flasks with 50 mL of medium.

### Protein purification (enzyme screen)

Cells were pelleted by centrifugation at 4000 × *g* for 10 min and then resuspended in 400 µL of lysis buffer containing 20 mM sodium phosphate, 1 mM MgCl_2_, 20 mM imidazole, 500 mM NaCl, 1 mM phenylmethanesulfonyl fluoride, 2 mg/mL lysozyme (Fluka), and 0.2 mg/mL DNAse I (Roche), adjusted to pH 7.4). The cell suspension was incubated at 30 °C for 30 min, after which lysis was completed by three cycles of freezing at −80 °C and thawing at 30 °C. Lysates were then loaded onto 96-well format Co^2+^-charged TALON affinity columns (GE Healthcare) and washed twice with 20 column volumes of washing buffer containing 20 mM Na phosphate, 500 mM NaCl, and 20 mM imidazole, adjusted to pH 7.5. Proteins were subsequently eluted from the column with elution buffer containing 20 mM Na phosphate, 500 mM NaCl, and 500 mM imidazole, adjusted to pH 7.5, which was later replaced with storage buffer (2 mM Tris HCl, 1 mM MgCl_2_, pH 7.4) using 96-well ultrafiltration plates with a 10 kDa molecular weight cutoff. Proteins were stored at 4 °C for no more than 1 day before conducting activity assays. As detailed below, for each purified protein, we recorded its concentration and assessed whether the expression strain could grow (Supplementary Fig 1 and Supplementary Data 8).

### Quantification of cell growth and protein concentration

Cell growth was quantified by measuring absorbance at 595 nm of the expression cultures prior to harvest. For this, 50 µL of overnight culture was diluted with 100 µL of saline (0.9% NaCl, 1 mM MgCl_2_) in 96-well clear flat-bottom plates, ensuring absorbance values were maintained below 1. Protein yield was quantified by adding 5 µL of purified protein solution to 150 µL of Bradford reagent (Biorad). After incubation at room temperature for 10 min, absorbances were measured at 590 and 450 nm, respectively. The ratio of A_590_ over A_450_ was found to be proportional to the protein concentration over a wide range^48^. Quantification was performed using a dilution series of bovine serum albumin (BSA) as a standard.

### Fed-batch fermentations for metabolite extract preparation

The fermentation vessel used for the cultivation of *E. coli* K-12 BW25113 had a nominal volume of 2.4 L (Bioengineering). It was equipped with a 3-level radial flow impeller (diameter = 3 cm) operated at 1,000 rpm and was aerated with sterile air at a rate of 1 L gas/L liquid/min while maintaining an operating pressure of 1.2 bar. The pH was set to 7.0 and controlled through the addition of 28% (w/v) NH_4_OH or 5 M HCl, and the temperature was kept constant at 37 °C. For glucose minimal medium fermentation, the initial medium contained 5 g/L glucose, 14.8 g/L KH_2_PO_4_, 4.44 g/L (NH_4_)_2_HPO_4_, 1.9 g/L Na citrate, 1.33 g/L MgSO_4_, 5 mg/L thiamine·HCl, and 9 mL/L of a trace element solution (0.18 g/L ZnSO_4_·7 H_2_O, 0.12 g/L CuCl_2_·2 H_2_O, 0.12 g/L MnSO_4_·H_2_O, and 0.18 g/L CoCl_2_·6 H_2_O)^49^. The initial medium for glycerol complex medium fermentation contained 3 g/L glycerol, 12 g/L Bactro tryptone, 24 g/L Bacto yeast extract, 2.2 g/L KH_2_PO_4_, and 9.4 g/L K_2_HPO_4_. Polypropylene glycol was added as required to mitigate foam formation. After inoculating 750 mL of the initial medium with 50 mL of overnight preculture grown in LB medium, a feed medium with increasing substrate concentration was added at a constant rate of 2 mL/min. This feed medium mirrored the initial medium composition, featuring 10-25-100 g/L glucose for the glucose fermentation and 6-15-60 g/L glycerol for the glycerol fermentation. The low-concentration feed was supplied for 12 h, the medium-concentration feed for an additional 4 h, and the high-concentration feed was continued until the fermentations were ceased, which occurred at an optical cell density at 600 nm of 17 for the minimal medium batch and 22 for the complex medium batch.

### Preparative metabolite extraction

Fifty-mL aliquots of fermentation broth were transferred to 50 mL tubes directly from the bioreactor and pelleted by fast centrifugation at 14,000 rpm for 1 min at 0 °C. The dry cell weight (DCW) in each tube was estimated from the culture OD, using a coefficient of 0.38 mg DCW/OD/mL. After the supernatant was discarded, the pellets were flash-frozen in liquid nitrogen. Each pellet was then extracted with 20 mL of 60:40% (v/v) ethanol:water solution at 80 °C for 10 min with occasional vortexing. All extracts were pooled, and the samples were dried under vacuum using a SpeedVac (Christ). The extracted metabolites were resuspended in water to achieve physiological concentration, considering a coefficient of 1.63 µL of intracellular fluid per mg of DCW^50^. Aliquots of the extracts from both fermentations were stored separately at −80 °C prior to further use.

### Nontargeted dynamic enzyme activity assays

The assay mixture for each enzyme consisted of 68.25 µL of assay buffer (2 mM Tris·HCl, 2 mM MgCl_2_, pH 7.5), 9.75 µL of a 10-fold concentrated cofactor mix (which included 1 mM of each ATP, GTP, UTP, TTP, CTP, ITP, NADH, NAD^+^, NADPH, NADP^+^, FAD, glutathione, CoA, acetyl-CoA, SAM, and tetrahydrofolate), 9.75 µL of *E. coli* metabolite extract obtained from glucose minimal medium cultures, and 9.75 µL of *E. coli* metabolite extract obtained from glycerol complex medium cultures. Assay mixtures were pre-warmed for 5 min at 37 °C, after which assays were initiated by adding 2.5 µL of purified enzyme that had been stored on ice. At twelve time points, 5 µL aliquots of the reaction mixture were sampled and mixed with 95 µL of a quenching solution (75% (v/v) methanol, 25% (v/v) assay buffer, chilled on dry ice) to halt the reaction. Using eight-channel pipettes, eight enzyme assays were performed simultaneously. After completing the eight assays, the 96-well plate containing the quenched reaction solutions was sealed and stored at −80 °C in preparation for mass spectrometric analysis.

### Flow injection time-of-flight mass spectrometry

The analysis of quenched enzyme assay samples was conducted using a platform comprising a Hitachi L-7100 liquid chromatography pump, a Gerstel MPS2 autosampler, and an Agilent 6550 IonFunnel QTOF mass spectrometer (Agilent, Santa Clara, CA) operated under established settings^37^. The isocratic flow rate was maintained at 150 μL/min, using a mobile phase of isopropanol and water (60:40, v/v) buffered with 5 mM ammonium fluoride at pH 9 for negative ionization mode. For online mass axis correction, 2-propanol (present in the mobile phase), taurocholic acid, and Hexakis(^1^H, ^1^H, ^3^H-tetrafluoropropoxy)phosphazine were used. Mass spectra were recorded in profile mode across 50 to 1000 m/z at a frequency of 1.4 spectra/s, utilizing the highest available resolving power of 4 GHz. The source temperature was set to 325 °C, with a drying gas flow rate of 5 L/min and a nebulizer pressure of 30 psig. Fragmentor, skimmer, and octupole voltages were set to 175 V, 65 V, and 750 V, respectively.

### Spectral data processing and annotation

All steps of mass spectrometry data processing and analysis were conducted using Matlab R2022a (The Mathworks, Natick) as described previously^37^. Briefly, peak detection was performed on each sample using the total profile spectrum obtained by summing all individual scans recorded over time, utilizing wavelet decomposition provided by the bioinformatics toolbox. During this procedure, a cutoff was applied to filter out peaks with fewer than 5000 ion counts in the summed profile spectrum, thereby avoiding the detection of artifacts. The centroid lists from samples were then merged into a single matrix by binning the accurate centroid masses within the tolerance defined by the instrument’s resolution. The resulting matrix detailed the intensity of each mass peak across all analyzed samples. An accurate common *m/z* was recalculated using a weighted average of the values obtained from independent centroiding. After merging, ions were annotated based on their accurate mass with a tolerance of 0.005 Da. Annotation was performed with the assumption that [M-H]^-^ and [M + F]^-^ were the dominant ions in negative mode. Additionally, several commonly observed abiotic adducts were annotated, including ^12^C-> ^13^C, H->Na, H-> K, and +NaCl. In this process, we used the KEGG *E. coli* compound database^36^ as a reference for annotation.

### Data processing and standardization

The raw dataset consisting of a matrix of intensities values for 9554 detected ions across 12,672 samples was processed as follows. (i) A median filter subtraction was applied to subtract temporal trends for each ion at each time point, using chronologically sorted enzyme assays to correct for instrument sensitivity drifts and other systematic temporal trends. (ii) Ion abundances were log_2_-normalized to the median abundance of each ion at each time point across all enzyme assays. This step was crucial for ensuring equal weight was assigned to both accumulating and decreasing ions. (iii) Iterative bi-directional Z-score (Eq. 1) standardization involved two phases. First, the abundance of each ion in each enzyme assay was normalized relative to its abundance across all other enzyme assays and time points. Second, the abundance of each ion in a specific enzyme assay was normalized to the abundance of all other ions within the same assay. This process was performed iteratively for a total of five iterations. (iv) Individual outliers, such as those arising from pipetting errors, were corrected through one-dimensional median filtering of each temporal ion profile in the respective enzyme assay. The effects of these data processing steps are further explained and visualized in Supplementary Fig 2.

For an ion $i$ in enzyme assay $j$ at time point $t$, the iterative bi-directional Z-score normalization was calculated iteratively as:

1 $${Z}_{i,j,t}^{(n+1)}=\frac{{Z}_{i,j,t}^{(n)}-{\mu }_{j,t}^{(n)}}{{\sigma }_{{j,t}^{(n)}}}, \, {{\rm{where}}} \, {Z}_{i,j,t}^{(0)}=\frac{{X}_{i,j,t}-{\mu }_{i}}{{\sigma }_{i}}$$

${X}_{i,j,t}$ is the abundance of ion $i$ in enzyme assay $j$ at time point $t$; and ${\mu }_{i}$ and ${\sigma }_{i}$ are the mean and standard deviation of ion $i$ across all enzyme assays and time points. Furthermore, ${\mu }_{j,t}^{(n)}$ and ${\sigma }_{j,t}^{(n)}$ are the mean and standard deviation of ${Z}_{i,j,t}^{(n)}$ across all ions within enzyme assay $j$ at time point $t$. The calculation is iterated five times (*n* = 0, 1, …,) and final Z-score is defined by the last iteration (*n* = 4).

### Quantification of metabolite ion responses and curve fitting

The average Z-score (Z_av_) for each ion in each assay was calculated as the arithmetic mean of the Z-scores at the last five time points (Eq. 2).

2 $${Z}_{{{av}}_{i,j}}=\frac{{\sum }_{{t}=8}^{12}{Z}_{i,j,t}}{5}$$

The rank product (RP) was determined as the geometric mean of the Z-score-based ranks of an ion’s abundance in an enzyme assay at the last five time points, compared to its abundance across all other ions at those same time points (Eq. 3). Ranking was conducted in two ways: ascending (where small Z-scores, representing substrates, receive low ranks) and descending (where large Z-scores, representing products, receive low ranks). Because both rankings are symmetric, only the ranking yielding the lowest rank product for an ion was used. To indicate low relative abundance, ascending ranks were multiplied by -1. Further details on the rank product method can be found in previous studies^39,51^.

3 $${{RP}}_{i,j}=\root{{5}}\of{{\prod }_{{t}=8}^{12}{{\mathrm{rank}}}_{i,j,t}}$$

Finally, we fitted a hyperbolic tangent (sigmoid) function to the temporal profile of each ion in each enzyme assay. This function is defined by two parameters: *A* (equilibrium) and *B* (sigmoid steepness) (Eq. 4):

4 $${Z}_{i,j,t}=A\cdot \tanh \left(\frac{B \cdot t}{\left|A\right|}\right)$$

We constraint *B* to be a positive number, while *A* could be either positive or negative, reflecting whether the ion is a substrate (*A* < 0) or a product (*A* > 0) in the measured assay. We divide *Bt* by the absolute value |*A*|, so that the derivative at *t* = *0* would be equal to *B* or *-B* (depending on the sign of *A*). Minimal least squares curve fitting was done using the lsqcurvefit function in MATLAB.

### Machine learning for hit identification

We trained a random forest classifier to identify ions that exhibited greater similarity to known reactant ion traces compared to noise. To train the classifier, we focused exclusively on ions that had at least one annotation for a deprotonated ion [M-H]^-^ of carbohydrates (i.e., those with formulas that include C and H), referred to as high confidence annotations. Positive control examples were defined as ion-enzyme pairs where the [M-H]^-^ annotation of the ion matched a known substrate or product and where the corresponding enzyme was expressed at measurable levels (Supplementary Fig 1 and Supplementary Data 8). Negative control examples included all ions from negative control experiments that did not contain enzymes, as well as ion traces not corresponding to known reactants in enzyme assays with undetectable enzyme levels. The parameters for the random forest model were tuned by splitting the dataset in a train (80%) and test set (20%). The optimal parameters were by taking the parameters with across five replicate trainings showed the lowest averaged out-of-bag prediction accuracies—i.e., the accuracies to predict each point only with trees that do not use the specific point for training. The parameter ranges and optimal values were as follows: maximum tree depth: 2-34 (optimal 5), maximum features: 10–100% (optimal 20%), criterion: gini versus entropy (optimal: entropy), and class weight: true versus false (optimal false). The number of trees was chosen to be sufficiently high that the out-of-bag accuracy was observed to be stable (*n* = 10,000). The classifier using the optimal, chosen parameters were also applied to the test set and a similar accuracy and f1 score was observed. For prediction, a classifier was trained using the full dataset, with uncalibrated prediction class probability serving as the readout. For points used for training, the out-of-bag prediction probabilities were used to minimize biases. Cutoffs for the prediction probabilities were established by calculating an upper bound for the False Discovery Rate^52^ based on the False Positive Rate (FPR), the total number of ions considered (n), and the number of hits exceeding each cutoff (*n*_co_). The FPR_co_ was estimated as the fraction of negative controls that passed each cutoff.

5 $${\mathrm{FDR}}_{\mathrm{thresh}}=({\mathrm{FDR}}_{\mathrm{co}} \cdot {n})/{n}_{\mathrm{co}}$$

A threshold corresponding to prediction probability with an FDR < 0.333 and the highest recall was designated as a “hit” threshold (p_pos > 0.3527). The same classifier was subsequently applied to all annotated ions, and the same “hit threshold” was enforced, resulting in a higher FDR (FDR = 0.890).

### Pathway enrichment analysis: reactant- and enzyme-centric

To identify pathways significantly associated with novel promiscuous reactants, we utilized the MetaboAnalyst platform^53^. The analysis was conducted using the hypergeometric test for over-representation, coupled with relative betweenness centrality for pathway topology assessment. The input comprised the frequency of metabolites identified as novel reactants from high-confidence ion traces, with the background set defined by all annotated ions detected in our mass spectrometry approach. Complementarily, we performed an enzyme-centric pathway enrichment analysis using ShinyGO^54^, applying Fisher’s exact test to assess over-representation of gene sets in functional categories. Enzymes associated with unexpected reactant changes were mapped to *E. coli* KEGG pathways, and enrichment was tested against the full set of successfully purified enzymes as background. No pathways passed significance (*p* < 0.05) after multiple-testing correction, indicating no detectable clustering of promiscuous enzymes in particular metabolic routes under our conditions.

### Protein purification (validation experiments)

Cells were pelleted by centrifugation at 4000 × *g* for 10 min and then resuspended in 4 mL of lysis buffer (100 mM Tris·HCl, 5 mM MgCl_2_, 2 mM dithiothreitol, 4 mM phenylmethanesulfonyl fluoride, pH 7.5). The mixture was cooled on ice, and lysis was performed by three passages through a FrenchPress system (Simo Aminco) using pressure cells precooled to 4 °C and operated at 1000 psig. The resulting cell lysates were applied to Co^2+^-charged TALON affinity columns (0.5 mL column volume, GE Healthcare), which achieve higher purification than conventional Ni^2+^-charged resins. The columns were washed twice with 20 column volumes of washing buffer (20 mM Na phosphate, 500 mM NaCl, 20 mM imidazole, pH 7.5). Step gradient elution was then performed with elution buffer (20 mM Na phosphate, 500 mM NaCl, pH 7.5) containing imidazole concentrations of 60, 100, and 500 mM. Fractions containing the target protein, identified by SDS-PAGE, were selected for further purification.

The buffer of the selected fractions was exchanged to 10 mM Tris·HCl (pH 7.5) through three ultrafiltration steps using spin columns with 10 kDa molecular weight cutoff (Millipore). In a second purification step, eluates from the affinity purification were applied to an anion exchange column packed with 1.5 mL Q-Sepharose High Performance resin (GE Healthcare), which was pre-loaded with Cl^-^ ions. Proteins were eluted in 4 mL fractions using a step gradient of 100, 200, and 500 mM NaCl in 20 mM phosphate buffer at pH 7.5, supplemented with 1 mM MgCl_2_, at a flow rate of 1 mL/min. Fractions containing the target proteins, as determined by SDS-PAGE, were pooled and concentrated to a final volume of 1 mL in buffer containing 100 mM Tris·HCl (pH 7.5) and 10 mM MgCl_2_ using ultrafiltration spin columns (Millipore) with a molecular weight cutoff of 10 kDa.

### Validation enzyme assays with pure substrates

Purified enzymes were incubated at 37 °C at a protein concentration of 50 µg/mL in a total of 150 µL buffer containing 10 mM Tris·HCl (pH 7.5), 1 mM MgCl_2_, and 10 mM of each substrate. The compounds were purchased from Sigma Aldrich at the highest available purity. At the specified time points, 10 µL of the reaction solution was transferred to 30 µL methanol that had been cooled with dry ice to quench the reaction by enzyme denaturation. The concentrations of reactants were subsequently measured by time-of-flight mass spectrometry^33^. Each experiment was performed at least in duplicate and included at least two independent enzyme purifications. As negative controls, heat-inactivated (treated at 95 °C for 60 min) proteins were used.

### Gel electrophoresis (SDS-PAGE)

Gel electrophoresis was conducted using precast 12-well, 1 mm thick NuPAGE 4–12% Bis-Tris Gels (Novex) together with premixed NuPAGE MOPS SDS running buffer (Novex). The gels were run at a constant voltage of 100 V with a PowerPac 1000 power supply (Biorad). After electrophoresis, gels were stained in a solution of 50:40:10% (v/v) methanol, water, and glacial acetic acid containing 2 g/L Coomassie Brilliant Blue R-250 at room temperature for 3 h. They were then destained in the same solution without the Coomassie dye until background remained negligible and finally digitalized using a scanner (Hewlett-Packard).

### Reporting summary

Further information on research design is available in the Nature Portfolio Reporting Summary linked to this article.

## Supplementary information

Supplementary Information

Supplementary Data 1-8

Description of Additional Supplementary Files

Reporting Summary

Transparent Peer Review file

## Data Availability

Raw mass spectrometry was made public through massive.ucsd.edu and can be accessed through 10.25345/C5MW28T8R^55^. Processed and normalized mass spectrometry data are published via 10.5281/zenodo.17242926^56^. Data tables and additional information required to perform analyses can be found on 10.5281/zenodo.17242926^56^.

## Code availability

Scripts and code for curve fitting, feature extraction, machine learning and key analyses were made public on 10.5281/zenodo.18881970^57^.
