# Supplementary material for: Systematic discovery of enzyme promiscuity in Escherichia coli using in vitro metabolomics
Source: Commun Biol. 2026 May 12;9:1004. doi: 10.1038/s42003-026-10099-x (PMC13389410; doi:10.1038/s42003-026-10099-x)
Supplement: Supplementary file 1 — Supplementary Information [file 42003_2026_10099_MOESM1_ESM.pdf]

Supplementary Figures for

Systematic discovery of enzyme promiscuity in *Escherichia coli* using *in vitro* metabolomics

Table of contents

Supplementary Figure 1 | Expression strain growth yields and purified protein concentrations.
Supplementary Figure 2 | Data processing and standardization.
Supplementary Figure 3 | Reaction prediction based on main reaction pairs from the KEGG database.
Supplementary Figure 4 | Experimental validation of the promiscuous reactions catalyzed by CobC.
Supplementary Figure 5 | Experimental validation of the promiscuous reactions catalyzed by NanK.
Supplementary Figure 6 | Experimental validation of the promiscuous reactions catalyzed by DeoA.
Supplementary Figure 7 | Experimental validation of the promiscuous reactions catalyzed by RihB.
Supplementary References

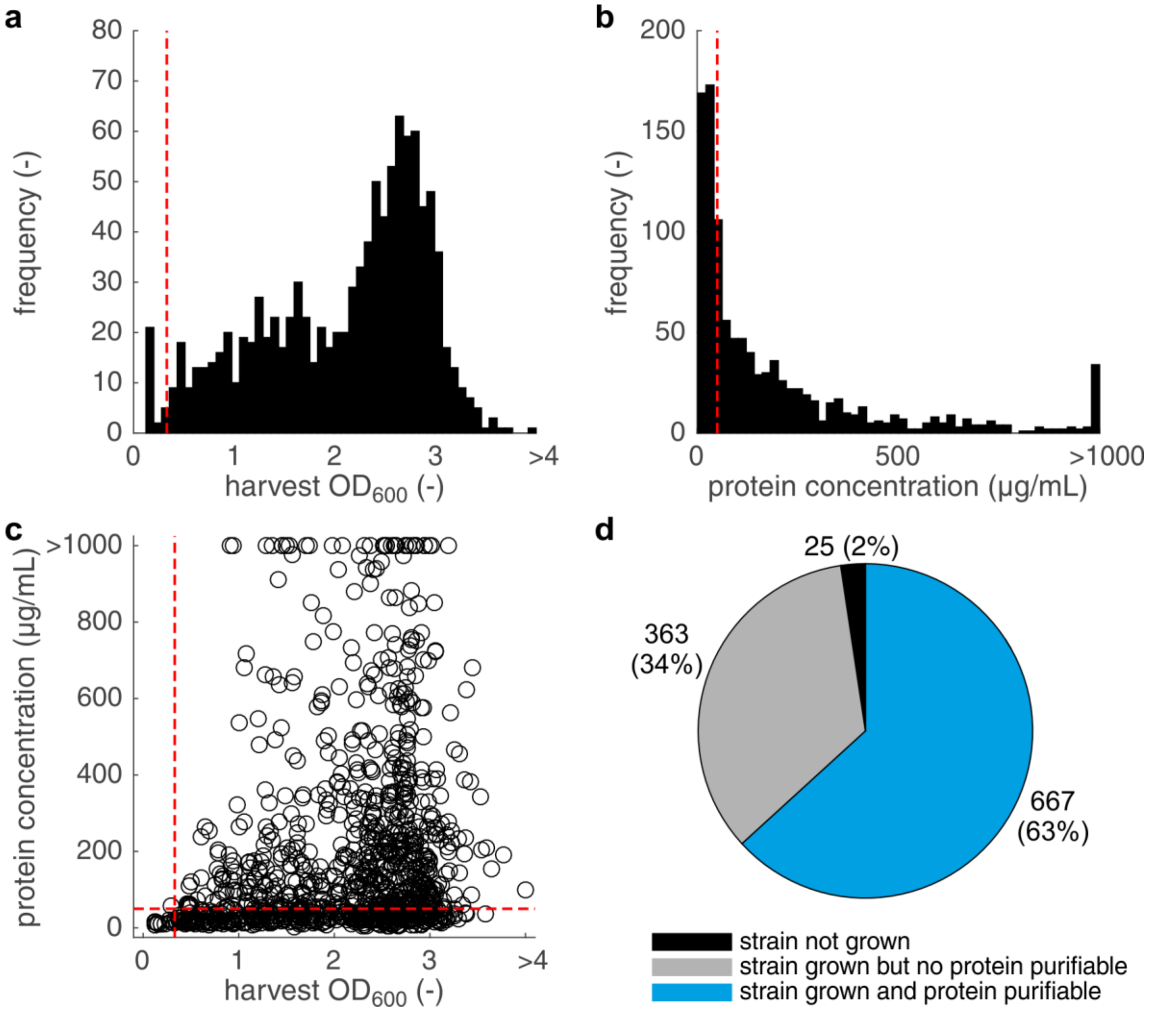

Supplementary Figure 1 | Growth yields of expression strains and concentrations of purified proteins. (**a**) Histogram of OD_{600} values of expression cultures at harvest. Strains that did not reach a minimum OD_{600} of 0.33 were excluded from further analyses. (**b**) Histogram of purified protein concentrations as determined by Bradford assays. Proteins with concentrations below 50 µg/mL were excluded from further analyses. (**c**) Scatter plot of protein concentrations versus harvest OD_{600}. Red lines indicate the cutoff values. (**d**) Summary of the numbers of proteins excluded due to low harvest OD_{600} or protein yield.

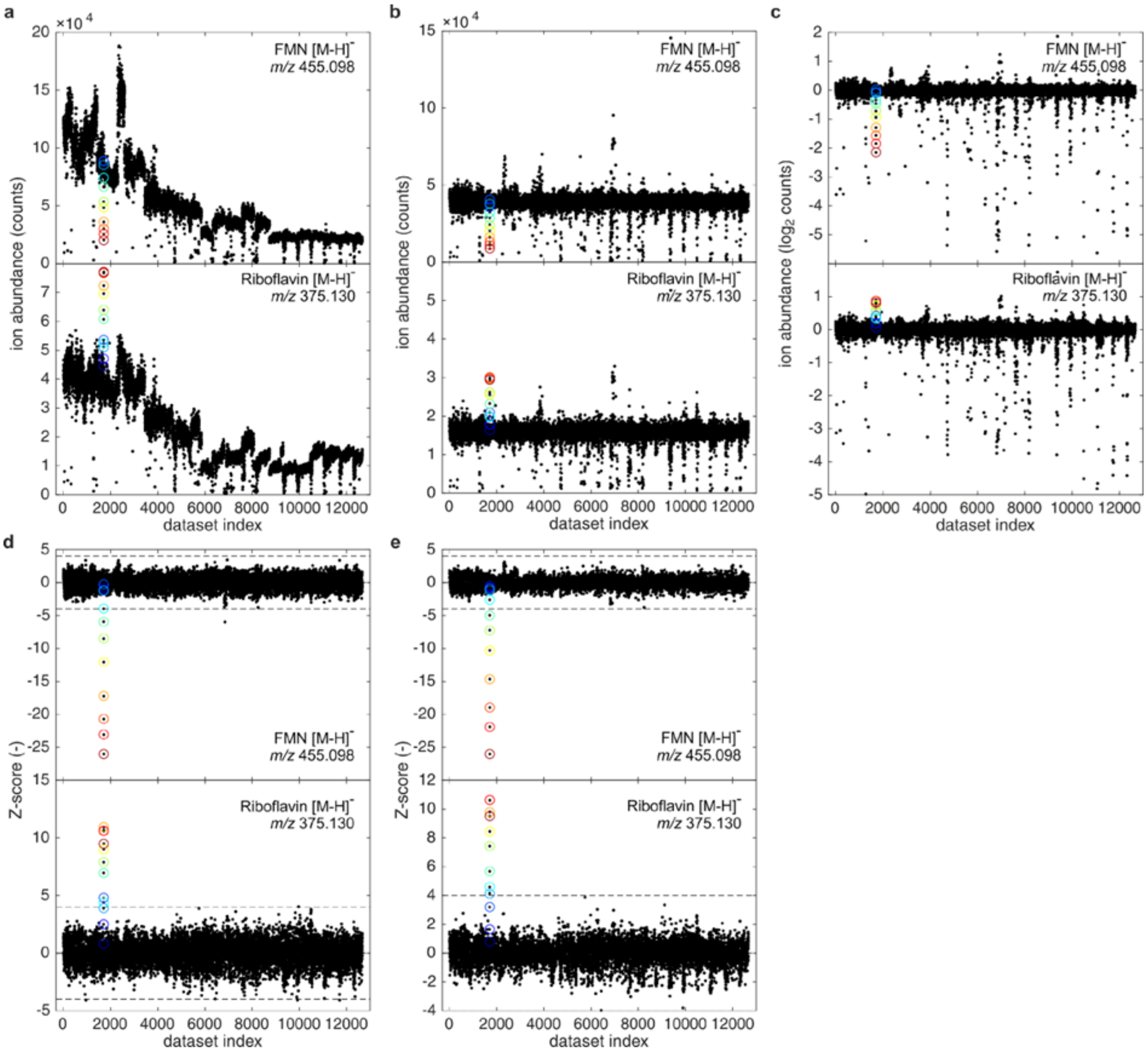

Supplementary Figure 2 | Data processing and standardization. All panels exemplarily refer to the ions *m/z* 455.098, annotated as flavin mononucleotide (FMN), and *m/z* 375.130, annotated as riboflavin. Datasets corresponding to assays of CobC, an enzyme found to convert FMN to riboflavin, are highlighted according to their time points. (**a**) Raw ion intensity. Main problems are high inter- and intra-day variance, which obscure biologically relevant effects, as well as the arbitrary measure of ion abundance, which varies between ions. These biases were corrected by subtracting a median filter of window size 10 from the abundance of each ion separately for each measurement batch. (**b**) Median-filtered data. To assign equal weight to depletions and accumulations, the abundance of each ion was $\log_2$-transformed. (**c**) Log_2-transformed data. Remaining problems are large variance among individual datasets caused by experimental inaccuracies and the arbitrary abundance measure for each ion. To allow systematic comparisons between different ions and to correct for individual outliers, iterative bi-directional *Z*-score standardization was applied to the abundance of each ion across all datasets.[1] (**d**) Standardized data. Residual outliers were corrected by applying a median filter of window size 3 to the time course of each ion in each data set. (**e**) Final *Z*-score data. The dashed line indicates the cutoff derived from known enzymes.

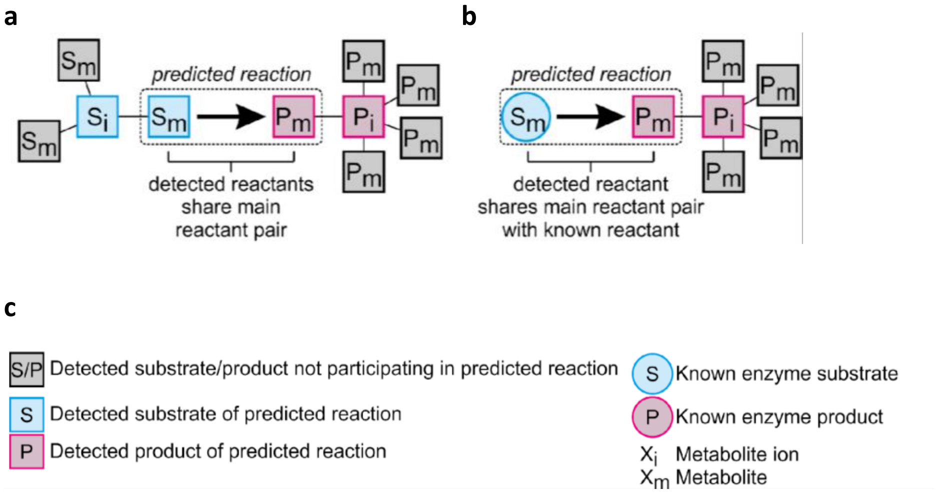

Supplementary Figure 3 | Reaction prediction based on main reaction pairs from the KEGG database. (**a**) Identification of reactant pairs by matching detected substrate and product metabolites to the KEGG main reactant pair list. (**b**) Identification of reactant pairs consisting of detected and known reactants of an enzyme. (**c**) Legend for panels b-e.

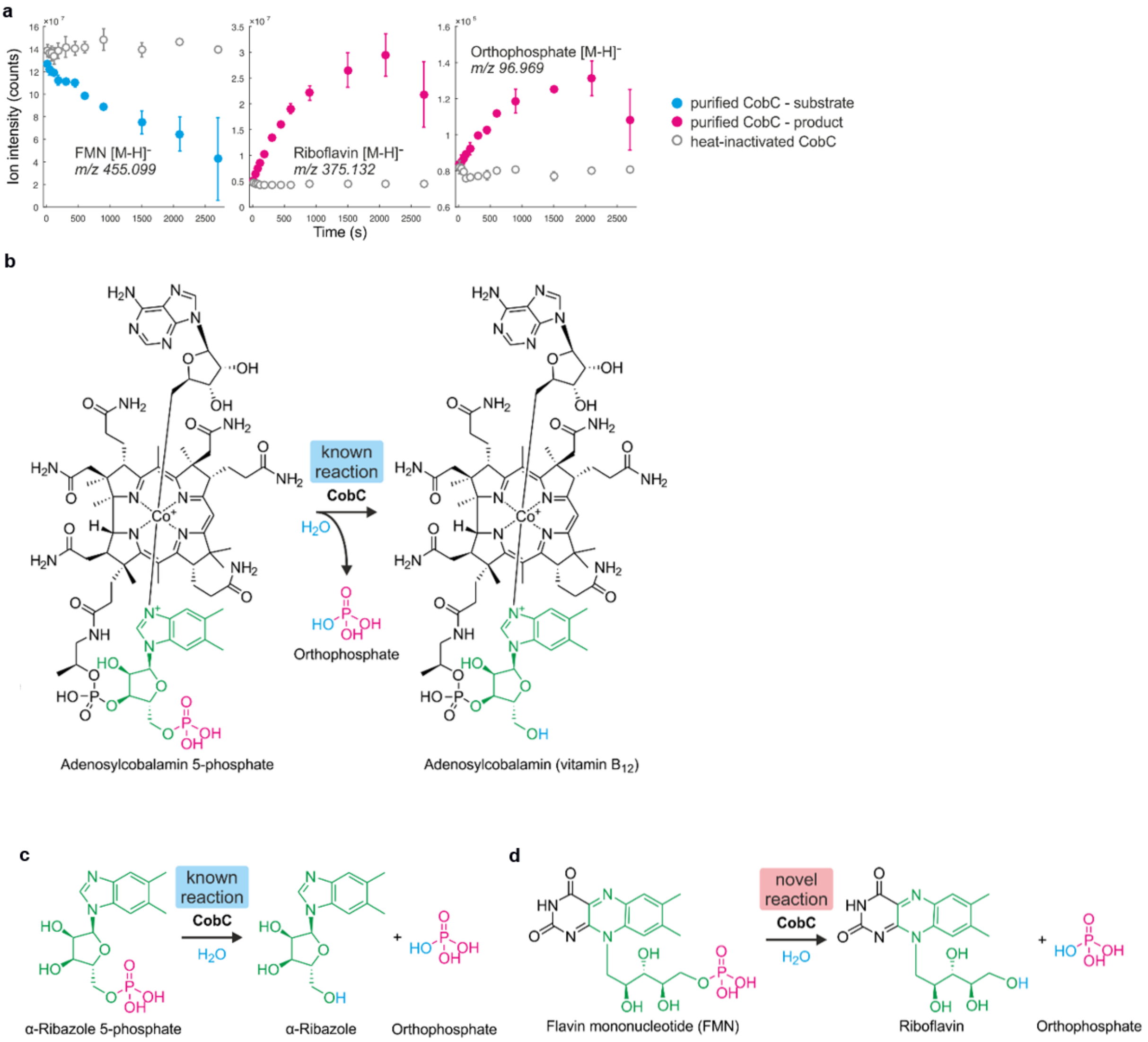

Supplementary Figure 4 | Experimental validation of the promiscuous reactions catalyzed by CobC. a: Mass-spectrometric abundance of detected reactant ions in assays with 50 µg/mL purified enzyme and 1 mM of each substrate. Data is shown as mean and standard deviation of 3 replicates. (**a**) Assay of purified CobC adding flavin mononucleotide (FMN) as substrate. Shown are mass-spectrometric abundances of detected reactant ions in assays with 50 µg/mL purified enzyme and 1 mM substrate. Data is shown as mean and standard deviation of 3 replicates. (**b**) Known reaction of CobC, the dephosphorylation of adenosylcobalamin 5-phosphate to adenosylcobalamine and orthophosphate. (**c**) Known reaction of CobC, the dephosphorylation of α-ribazole 5-phosphate to α-ribazole and orthophosphate. (**e**) Newly identified promiscuous reaction of CobC, the dephosphorylation of FMN to riboflavin and orthophosphate.

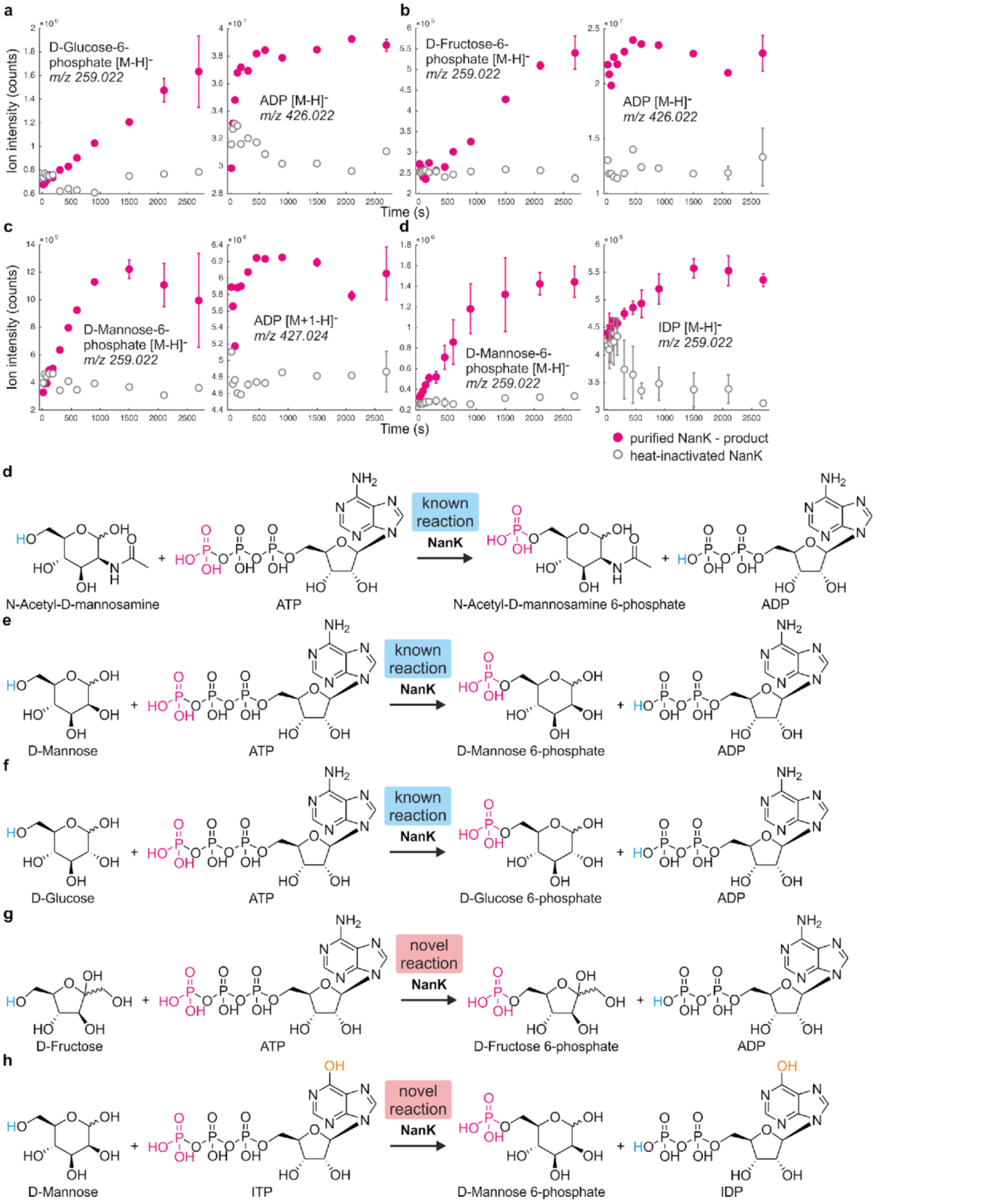

Supplementary Figure 5 | Experimental validation of the promiscuous reactions catalyzed by NanK. Panels a – d: Mass-spectrometric abundance of detected reactant ions in assays with 50 µg/mL purified enzyme and 1 mM of each substrate. Data is shown as mean and standard deviation of 3 replicates. (**a**) Substrates were D-glucose and ATP. (**b**) Substrates were D-fructose and ATP. (**c**) Substrates were D-mannose and ATP. (**d**) Substrates were D-mannose and ITP. (**d**) to (**f**) Known reactions of NanK, the ATP-dependent phosphorylation of N-acetyl-D-mannosamine, D-mannose and D-glucose yielding ADP as well as N-acetyl-D-mannosamine 6-phosphate, D-mannose 6-phosphate and D-glucose 6-phosphate, respectively. (**g**) and (**h**) Promiscuous reactions of NanK, the ATP-dependent phosphorylation of D-fructose to ADP and D-fructose 6-phosphate, as well as the ITP-dependent phosphorylation of D-mannose yielding IDP and D-mannose 6-phosphate.

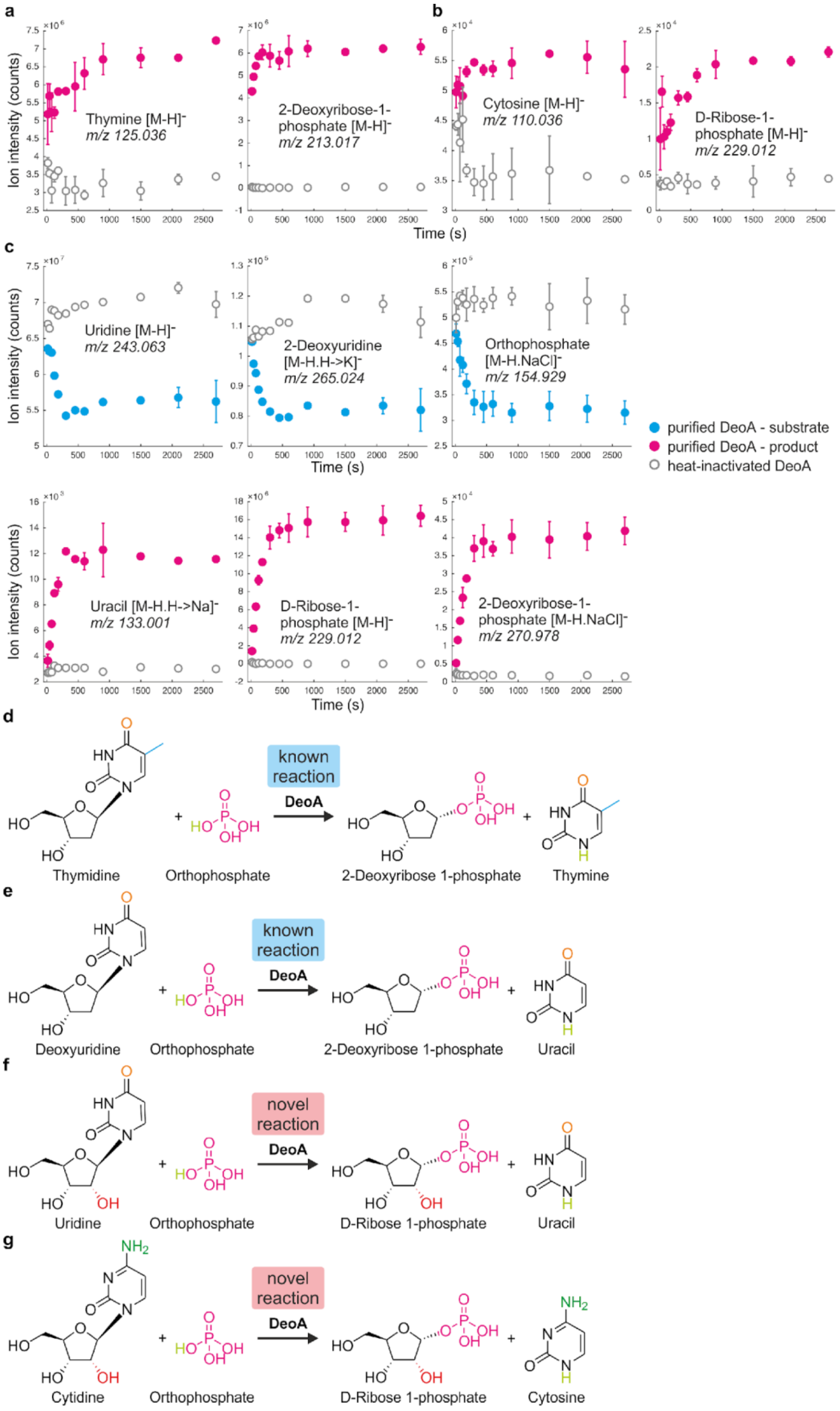

Supplementary Figure 6 | Experimental validation of the promiscuous reactions catalyzed by DeoA. Panels a – c: Mass-spectrometric abundance of detected reactant ions in assays with 50 µg/mL purified enzyme and 1 mM of each substrate. Data is shown as mean and standard deviation of 3 replicates. (**a**) Reaction with thymidine and sodium phosphate as substrates. (**b**) Reaction with cytidine and sodium phosphate. Cytidine was tested based on its structural similarity to the newly identified

substrate thymidine. (**c**) Reaction with uridine, deoxyuridine, and sodium phosphate. (**d**, **e**) Known reactions of DeoA: phosphorylytic cleavage of thymidine and deoxyuridine, yielding deoxyribose 1-phosphate along with thymine and uracil, respectively. (**f**) Promiscuous reaction of DeoA: phosphorylytic cleavage of uridine to ribose 1-phosphate, tested based on full stoichiometry reconstruction from the main screen. (**g**) Additional promiscuous reaction of DeoA identified using cytidine, tested due to its structural similarity to the novel substrate uridine.

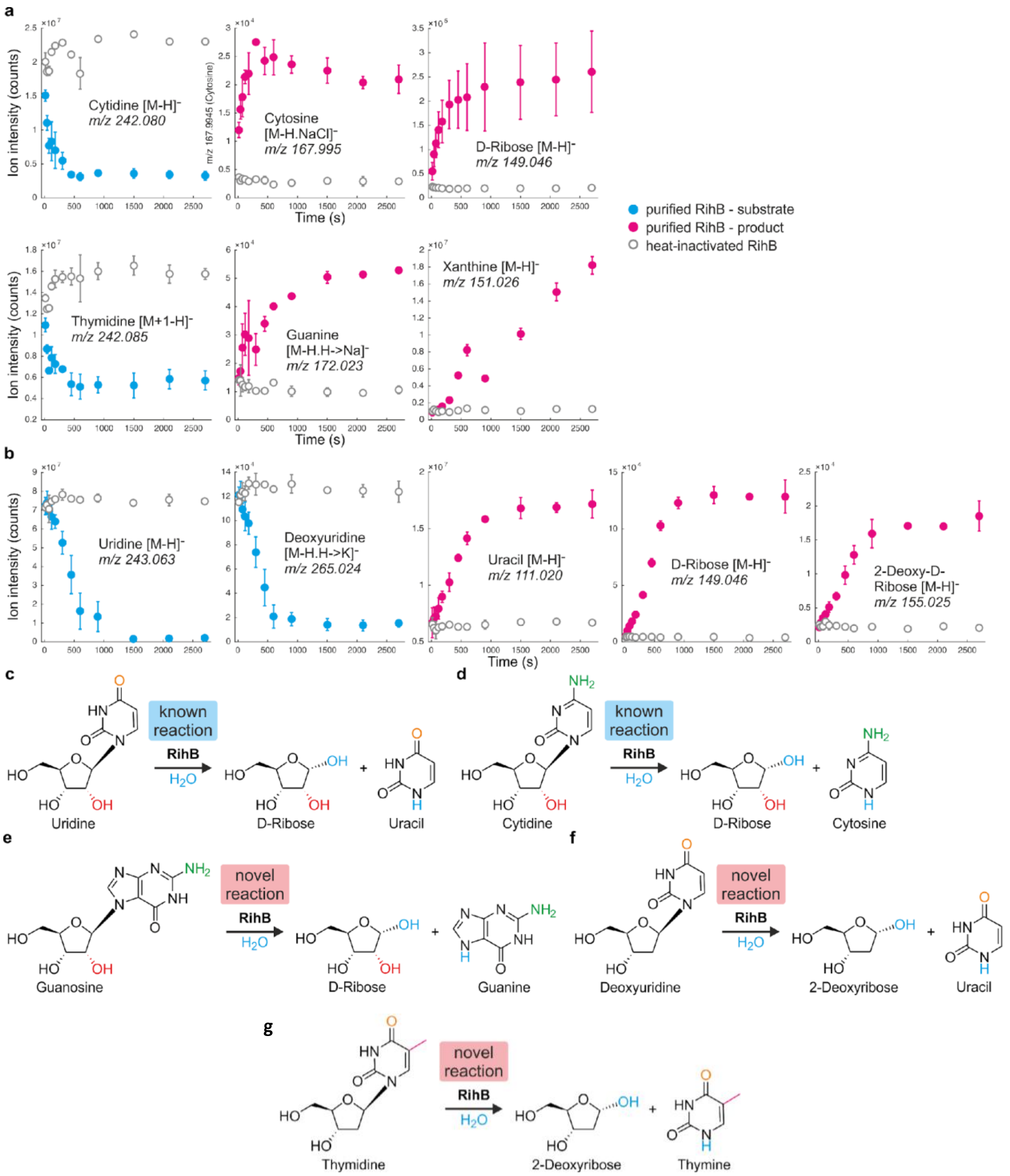

Supplementary Figure 7 | Experimental validation of the promiscuous reactions catalyzed by RihB. Panels a – b: Mass-spectrometric abundance of detected reactant ions in assays with 50 µg/mL purified enzyme and 1 mM of each substrate. Data is shown as mean and standard deviation of 3 replicates. (**a**) Substrates were cytidine, thymidine, guanosine and xynthosine. (**b**) Substrates were uridine and deoxyuridine. (**c**) and (**d**) Known reactions of RihB, the hydrolysis of uridine and cytidine to D-ribose as well as uracil and cytosine, respectively. (**e**) – (**g**) Promiscuous reactions of RihB, the cleavage of the (deoxy)nucleosides guanosine, deoxyuridine and thymidine yielding the separate (deoxy)ribose and free nucleobase moieties. Thymidine was predicted to be a substrate based on ion trace annotated as potassium thymidine adduct [.H/K-H(+)], guanosine and deoxyuridine were tested based on structural similarity to known substrates.

Supplementary references

1. Olshen, R. a. & Rajaratnam, B. Successive normalization of rectangular arrays: Rates of convergence. *Proc. - 1st Int. Conf. Data Compression, Commun. Process. CCP 2011* **38,** 239–245 (2011).
