# Supplementary material for: Systematic discovery of enzyme promiscuity in Escherichia coli using in vitro metabolomics
Source: Commun Biol. 2026 May 12;9:1004. doi: 10.1038/s42003-026-10099-x (PMC13389410; doi:10.1038/s42003-026-10099-x)
Supplement: Supplementary file 3 — Description of Additional Supplementary Files [file 42003_2026_10099_MOESM3_ESM.pdf]

Description of Additional Supplementary Files

File name: Supplementary Data 1

Description: Annotation of ions as metabolites based on matching their accurate mass with compounds in the KEGG eco database. Mass tolerance was 0.001 Da. Assumed ions and neutral gains and losses are indicated in square brackets.

File name: Supplementary Data 2

Description: A list of all ion traces declared as hits when considering only deprotonated ions at a 33% false discovery rate (FDR) threshold. It includes extracted parameters used in the machine learning approach, the resulting classifier scores, and whether each ion trace was previously reported in databases or metabolic models.

File name: Supplementary Data 3

Description: A list of all ion traces declared as hits when considering adducts and unannotated ions. The table includes extracted parameters used in the machine learning approach, the resulting classifier scores, and whether each ion trace was previously reported in databases or metabolic models.

File name: Supplementary Data 4

Description: Known catalyzed reactions of 91 test cases with unexpected reactants obtained from Ecocyc database, giving information on which enzymes were previously known to be promiscous versus which were reported to be specific.

File name: Supplementary Data 5

Description: Data for the metabolite pathway enrichment analysis, comparing the observed frequencies of reactants involved in novel promiscuous activities to their representation in the KEGG E. coli metabolite subset detected in our mass spectrometry measurements.

File name: Supplementary Data 6

Description: Complete reaction stoichiometries inferred from observed metabolite changes before manual curation. It includes information on detected substrates and products, their KEGG main reactant pair matches, and associated cofactors. Information on which reactions where manually excluded.

File name: Supplementary Data 7

Description: This table presents a manually curated set of promiscuous reactions, as well as information on new and known EC numbers to distinguishing substrate ambiguity from catalytic promiscuity. It includes enzyme assignments, EC number comparisons, and mechanistic insights into how these reactions expand enzymatic function. In contrast to table 6, this table was manually curated, removing some known reactions, as well as ambiguous or redundant ones.

File name: Supplementary Data 8

Description: All strains of the ASKA library used in this work for His-tagged protein expression and purification. The optical density at 600 nm (OD600) and concentration of purified protein (determined by Bradford assay) listed.
