# Supplementary material for: Systematic discovery of enzyme promiscuity in Escherichia coli using in vitro metabolomics
Source: Commun Biol. 2026 May 12;9:1004. doi: 10.1038/s42003-026-10099-x (PMC13389410; doi:10.1038/s42003-026-10099-x)
Supplement: Supplementary file 5 — Transparent Peer Review file [file 42003_2026_10099_MOESM5_ESM.pdf]

nature portfolio

Peer Review File

Systematic discovery of enzyme promiscuity in Escherichia coli using in vitro metabolomics

Corresponding Author: Professor Uwe Sauer

This file contains all reviewer reports in order by version, followed by all author rebuttals in order by version.

Version 0:

Reviewer comments:

Reviewer #1

(Remarks to the Author)
We thank the authors for their thoughtful comments to our previous questions. The new manuscript appears to be significantly scaled back to reflect the most conservative and confident conclusions. Based on this new scope, I have no significant questions or comments on the work. This seems to be ready to publish.

Open Access This Peer Review File is licensed under a Creative Commons Attribution 4.0 International License, which permits use, sharing, adaptation, distribution and reproduction in any medium or format, as long as you give appropriate credit to the original author(s) and the source, provide a link to the Creative Commons license, and indicate if changes were made.
In cases where reviewers are anonymous, credit should be given to 'Anonymous Referee' and the source.
The images or other third party material in this Peer Review File are included in the article's Creative Commons license, unless indicated otherwise in a credit line to the material. If material is not included in the article's Creative Commons license and your intended use is not permitted by statutory regulation or exceeds the permitted use, you will need to obtain permission directly from the copyright holder.
To view a copy of this license, visit https://creativecommons.org/licenses/by/4.0/
